# An interdisciplinary knowledge translation intervention in long-term care: Study protocol for the vitamin D and osteoporosis study (ViD*OS*) pilot cluster randomized controlled trial

**DOI:** 10.1186/1748-5908-7-48

**Published:** 2012-05-24

**Authors:** Courtney C Kennedy, George Ioannidis, Lora M Giangregorio, Jonathan D Adachi, Lehana Thabane, Suzanne N Morin, Richard G Crilly, Sharon Marr, Robert G Josse, Lynne Lohfeld, Laura E Pickard, Susanne King, Mary-Lou van der Horst, Glenda Campbell, Jackie Stroud, Lisa Dolovich, Anna M Sawka, Ravi Jain, Lynn Nash, Alexandra Papaioannou

**Affiliations:** 1 Department of Medicine, McMaster University, 1280 Main Street West, Hamilton, Ontario L8S 4K1, Canada; 2 Hamilton Health Sciences - St. Peter's Hospital, Juravinski Research Centre, 88 Maplewood Avenue, Hamilton, Ontario L8M 1W9, Canada; 3 Department of Clinical Epidemiology & Biostatistics, McMaster University, 1280 Main St. West, Hamilton, Ontario, L8S 4K1, Canada; 4 Charlton Medical Centre, 25 Charlton Ave East, Hamilton, Ontario, L8N 1Y2, Canada; 5 Department of Kinesiology, Faculty of Applied Health Sciences, University of Waterloo, 200 University Ave West, Waterloo, Ontario, N2L 3G1, Canada; 6 Department of Medicine, Division of General Internal Medicine, MUHC-Montreal General Hospital, 1650 Cedar Avenue, Montreal, QC, H3G 1A4, Canada; 7 Schulich School of Medicine & Dentistry, The University of Western Ontario, Parkwood Hospital, 801 Commissioners Rd. East, London, Ontario, N6C 5J1, Canada; 8 Department of Medicine, Division of Endocrinology and Metabolism, University of Toronto, St. Michael's Hospital, Toronto, Ontario, Canada; 9 Medical Pharmacies Group Limited, 590 Granite Court, Pickering, Ontario, L1W 3X6, Canada; 10 Department of Family Medicine, McMaster Innovation Park, 175 Longwood Road South, Hamilton, Ontario, L8P 0A1, Canada; 11 Department of Medicine, Division of Endocrinology and Metabolism, Toronto General Hospital, 200 Elizabeth Street, Toronto, Ontario, M5G 2C4, Canada; 12 Ontario Osteoporosis Strategy, Osteoporosis Canada, 1090 Don Mills Road, Suite 301, Toronto, Ontario, M3C 3R6, Canada

**Keywords:** Knowledge translation, Long-term care, Nursing home, Osteoporosis, Fractures, Vitamin D, Multifaceted, Interdisciplinary, Feasibility, Audit and feedback, Reminders, Interactive, Educational meeting, Opinion leader

## Abstract

**Background:**

Knowledge translation (KT) research in long-term care (LTC) is still in its early stages. This protocol describes the evaluation of a multifaceted, interdisciplinary KT intervention aimed at integrating evidence-based osteoporosis and fracture prevention strategies into LTC care processes.

**Methods and design:**

The Vitamin D and Osteoporosis Study (ViD*OS*) is underway in 40 LTC homes (n = 19 intervention, n = 21 control) across Ontario, Canada. The primary objectives of this study are to assess the feasibility of delivering the KT intervention, and clinically, to increase the percent of LTC residents prescribed ≥800 IU of vitamin D daily. Eligibility criteria are LTC homes that are serviced by our partner pharmacy provider and have more than one prescribing physician. The target audience within each LTC home is the Professional Advisory Committee (PAC), an interdisciplinary team who meets quarterly. The key elements of the intervention are three interactive educational sessions led by an expert opinion leader, action planning using a quality improvement cycle, audit and feedback reports, nominated internal champions, and reminders/point-of-care tools. Control homes do not receive any intervention, however both intervention and control homes received educational materials as part of the Ontario Osteoporosis Strategy. Primary outcomes are feasibility measures (recruitment, retention, attendance at educational sessions, action plan items identified and initiated, internal champions identified, performance reports provided and reviewed), and vitamin D (≥800 IU/daily) prescribing at 6 and 12 months. Secondary outcomes include the proportion of residents prescribed calcium supplements and osteoporosis medications, and falls and fractures. Qualitative methods will examine the experience of the LTC team with the KT intervention. Homes are centrally randomized to intervention and control groups in blocks of variable size using a computer generated allocation sequence. Randomization is stratified by home size and profit/nonprofit status. Prescribing data retrieval and analysis are performed by blinded personnel.

**Discussion:**

Our study will contribute to an improved understanding of the feasibility and acceptability of a multifaceted intervention aimed at translating knowledge to LTC practitioners. Lessons learned from this study will be valuable in guiding future research and understanding the complexities of translating knowledge in LTC.

**Trial registration:**

ClinicalTrials.gov NCT01398527.

## Background

The field of knowledge translation (KT) attempts to bridge the gap between the generation of research evidence and the application of this evidence into clinical practice. KT is commonly defined as ‘a dynamic and iterative process that includes synthesis, dissemination, exchange and ethically-sound application of knowledge’ [1]. Examining the effectiveness of KT strategies across different contexts, healthcare professions, and target behaviors is essential [2] if we are to effectively bridge the gap between research evidence and clinical practice. To guide decision makers in choosing the best implementation strategies, rigorous evaluations of KT programs, including well-designed cluster randomized trials, are needed [3].

Despite a growing body of KT evidence in acute care or community settings, KT research in long-term care (LTC) is still in its early stages [4-6]. Recent LTC initiatives have examined the role of organizational context and/or developed empirically-based theories related to KT in LTC [7-9]. However, few studies within the LTC setting have focused on evaluating the effectiveness of common behaviour change strategies (*e.g.*, audit and feedback, educational materials, reminders) [10,11], particularly those involving multifaceted interventions. In a recent scoping review by Bostrom *et al.*[5], only 3.6% (n = 61) of KT studies identified were related to older adults, and approximately one-half of them were done in LTC. Problematic is the fact that the majority of these studies were not targeted at the organizational level, did not report on system level outcomes, and included only a single KT strategy (*e.g.*, audit and feedback alone). The majority of KT interventions, regardless of setting, have not targeted the entire interdisciplinary team (*i.e.*, physicians, nurses, pharmacists, dietitians, rehabilitation therapists, and other professionals) [12]. Of the LTC studies in the Bostrom review [5], 60% did not target mixed professional groups despite the emphasis on collaboration among the disciplines practicing in LTC [13]. Previous multifaceted interventions for interdisciplinary teams have had some success within the LTC setting [14,15].

Implementing evidence into practice requires whole system change [2,16], particularly in the LTC setting [5,17]. Berta *et al.*[6] suggest the majority of factors that may enhance the uptake and use of evidence-based practices in LTC are organizational and include: a working culture that facilitates cooperation and knowledge exchange; standardization of activities; experienced clinical leaders that engage others in the process; and ultimately the incorporation of guidelines that are reinforced by regulatory bodies. Other factors that enhance implementation of evidence-based practices in LTC include strong leadership [17-19], medical directives, and building best practices recommendations into training materials [20].

### Identifying the knowledge-to-action gaps

The topic of our KT intervention is evidence-based osteoporosis management and fracture prevention strategies [21-27]. Previous research by our team indicates that many LTC providers are unaware of osteoporosis and fracture prevention best practices, or have concerns surrounding diagnosis and treatment in elderly patients [28,29]. Furthermore, as a recent survey of LTC Medical Directors and Directors of Nursing documented, barriers to fracture care were modifiable and could be overcome through education and changes to local care delivery systems [30]. Rather than focusing on individual providers, we propose a model that takes a more collective approach and emphasizes integrating evidence-based practices into care processes.

In addition to proper assessment of individuals at high risk for fracture [21], we are emphasizing the wide-scale implementation of adequate levels of vitamin D (≥800 IU/day) because it is a tolerable, low-cost intervention with strong evidence that it can prevent fractures and falls in LTC residents [22,24,26,27,31]. In an environmental scan of 15 LTC homes we conducted in 2008 (n = 3,132 residents), the overall rate of vitamin D use was 38% [32], and there was considerable variation between homes with rates ranging from 11 to 62%. Another study [33] we conducted using data collected via the Resident Assessment Instrument (RAI) 2.0 [34,35] found similar results.

We developed a multifaceted, interdisciplinary KT intervention to improve the use of evidence-based osteoporosis and fracture prevention practices in LTC homes. The current report outlines the research design and protocol for evaluating this KT intervention.

## Methods

### Study population

The Vitamin D and Osteoporosis (ViD*OS*) study is currently underway in 40 LTC homes (19 intervention and 21 control) in Ontario, Canada. In Canada, LTC homes (also known as nursing homes or homes for the aged) are government-regulated facilities designed for individuals who require onsite nursing care, 24-h supervision, or personal support [36]. Our recruitment strategy included LTC homes located in communities of all sizes and geographical regions across the province of Ontario.

In order to make this study as generalizable as possible, we have only two facility-level eligibility criteria, and no patient-level criteria. All LTC homes serviced by our partner pharmacy provider (Medical Pharmacies Group Limited) and who have more than one prescribing physician (at the time of recruitment) were eligible for recruitment into the study. Medical Pharmacies is a large pharmacy provider whose services include medication packaging and distribution, clinical support, and consulting services to approximately one-third of all LTC homes in Ontario. Our rationale for excluding LTC homes with only one treating physician is to maintain anonymity during the presentation of prescribing reports at educational sessions. Furthermore, the requirement of having at least two physicians per home decreases sample size as it contributes to a lower intracluster correlation coefficient [37].

### Aims and objectives

The aim of the ViD*OS* study is to evaluate the feasibility and effectiveness of a multifaceted KT intervention to better integrate evidence-based osteoporosis and fracture prevention care processes in LTC. In addition to measuring feasibility of the intervention, the primary clinical objective is to determine if the intervention can increase the proportion of residents who are prescribed adequate levels of vitamin D (≥800 IU/day). Secondary objectives include: to determine if the intervention increases the prescribing of calcium supplements (≥500 mg/day elemental calcium); to determine if the intervention increases the prescribing of osteoporosis medications in high-risk individuals (*i.e.*, documented osteoporosis or prior hip fracture); to understand the experience of the LTC team with the intervention and which components were perceived as feasible, acceptable, and effective; and to document falls and fractures occurring during the study period.

### Study outcomes

#### Feasibility

Feasibility outcomes are measured at the facility-level and include (some are only relevant to intervention homes): the proportion of homes that are recruited and retained, attendance of Medical Directors and other professionals at educational sessions, internal champions identified, action plan items identified and initiated, performance reports provided and reviewed, falls and fracture data collection completed. A criterion of ≥80% will be used as the criterion for success on each of these feasibility measures, with the exception of recruitment. Other cluster randomized controlled trials (RCTs) in nursing homes aimed at changing the behaviour of health professionals have noted recruitment in the 40 to 50% range [38-40], thus our recruitment criterion for success was 40%.

#### Clinical

**Figure 1:**
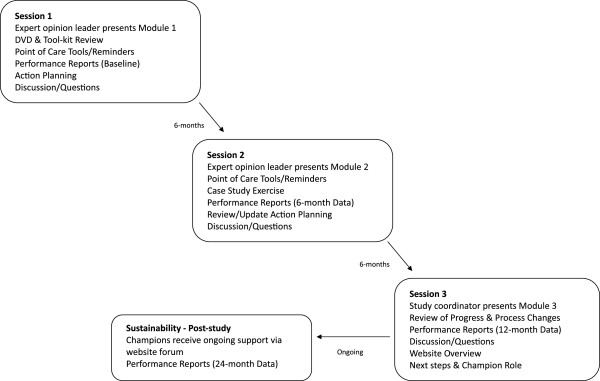
Overview of ViDOS Intervention.

The primary clinical outcomes are the proportion of residents prescribed vitamin D ≥800 IU/day at 6 and 12 months. The secondary prescribing outcomes are the proportion of residents prescribed ≥500 mg/day of elemental calcium and the proportion of high risk residents (*i.e.*, those with a fracture or documented osteoporosis) prescribed an osteoporosis medication (oral and IV bisphosphonates, teriparatide, denosumab) at 6 and 12 months. Other secondary outcomes include the number of falls, hip fracture and all fracture (hip, wrist, spine, foot, humerus, ribs, clavicle, ankle, other) for the data collection periods (*i.e.*, three months of falls and fracture data collected three times during the study, see Figure 1).

#### Study design

The ViD*OS* study is a pilot, cluster RCT [41] comparing a multifaceted KT intervention with a control group. The intervention is delivered over a 12-month time period with data collection extending to 16 months. Allocation by clusters of LTC homes rather than individual practitioners was chosen to minimize contamination because we are targeting interdisciplinary care teams. Because few other studies have examined this type of interdisciplinary multifaceted intervention within LTC, the study was designed as a pilot RCT that emphasizes feasibility outcomes [42].

#### Randomization and consent

Stratified block randomization was used to randomly allocate LTC homes to the intervention or control arm of the study (recruitment is now closed). LTC homes were stratified based on home size and profit/non-profit. Profit status was taken into consideration because there is some evidence that the quality of care is higher in non-profit homes compared to for-profit homes [43-45]. The allocation sequence was computer generated using nQuery 6.0 software by an off-site research member who is not involved in the recruitment, enrollment of clusters, or data analysis. Once the appropriate representative from the home was consented, the independent member assigned intervention and control groups based on the sequence and notified the coordinating centre. Because the homes are not blinded to treatment arms, the homes were informed of their allocation.

#### Intervention

A multifaceted strategy was chosen based on the consistent evidence that the most successful KT interventions tend to be interactive and multifaceted [3,10,11,14,15,46-49]. Systematic reviews of single interventions such as audit and feedback [50,51], reminders (*i.e.*, tools to aid decision-making and/or prompt a clinical action) [50], and opinion leaders [52] have also demonstrated some effectiveness in changing professional practice. As described below, we tailored our KT intervention to fit within the existing operational and organizational culture of each LTC home.

#### Target audience

The target audience of the multifaceted intervention is the Professional Advisory Committee (PAC), an interdisciplinary team [53] that meets quarterly to address resident care and quality improvement objectives. Members of the committee typically include: the Administrator, Medical Director, Director of Care, Consultant Pharmacist, Director of Food Services/Dietician, and other nursing, medical or rehabilitation staff. In addition to PAC team members, all physicians responsible for the care of residents within the LTC home are invited to the sessions and are eligible for continuing medical education credits with the Ontario College of Family Physicians.

#### Development and piloting the intervention

The multifaceted intervention was developed and piloted in consultation with the PAC team at a local LTC home. This home identified several procedural and organizational barriers that we addressed in the final version of our modules and materials. Learning modules and materials were built around a toolkit (including DVD, posters, panel cards, case studies) we developed for the *Ontario Osteoporosis Strategy in LTC* (http://www.osteoporosislongtermcare.ca[54]) in consultation with stakeholders. Materials were based on a research synthesis on hip fracture prevention strategies in LTC [22] and incorporate the 2010 Osteoporosis Canada Clinical Practice Guidelines [21].

#### Multifaceted intervention components

**Figure 2:**
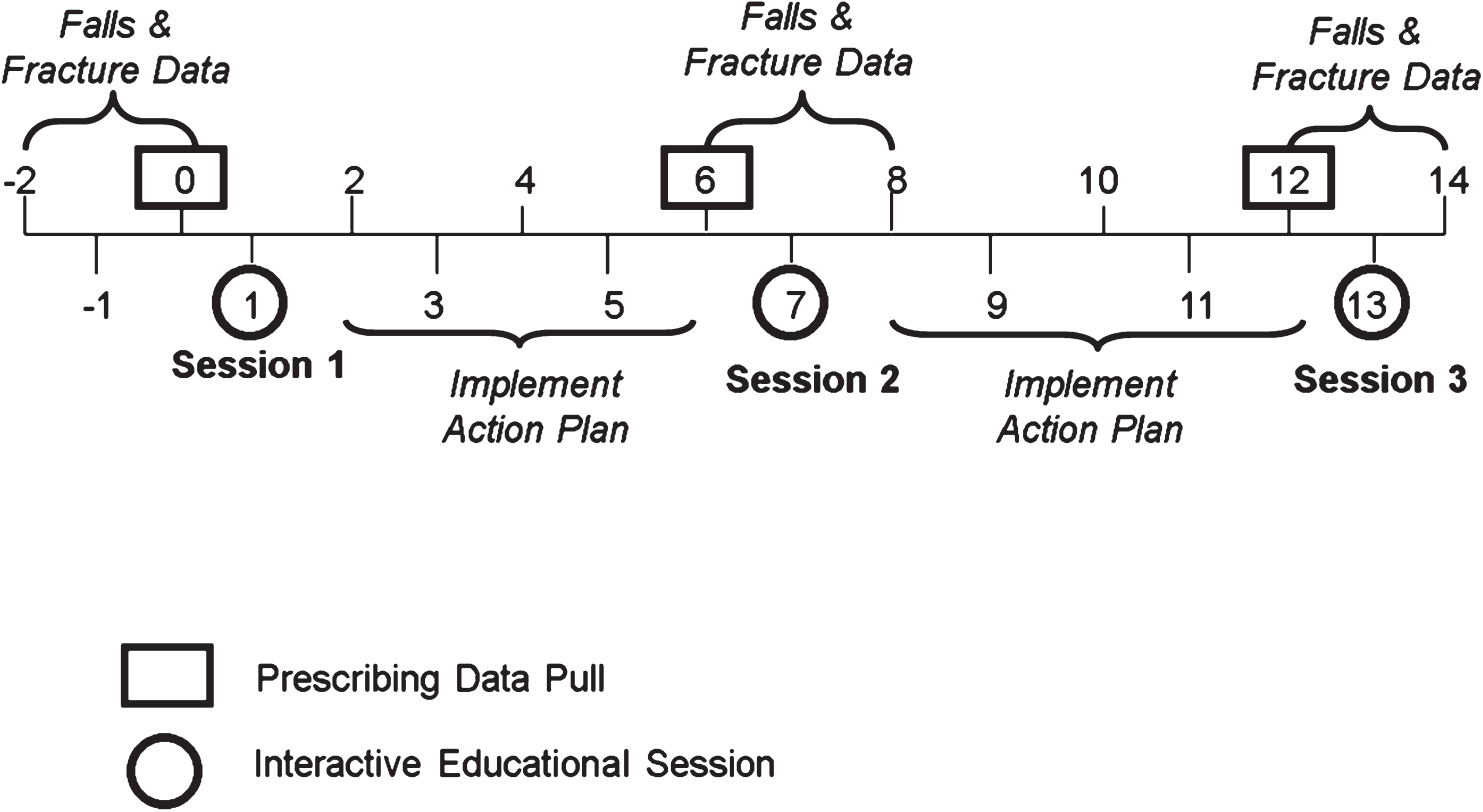
Timeline of Data Collection and Sessions.

As outlined in Figures 1 and 2, intervention homes take part in three interactive educational sessions, approximately six months apart. To maximize participation, these sessions are delivered during a regularly scheduled meeting of the PAC team. An expert opinion leader facilitates the first two interactive educational sessions (approximately 45 to 60 min in length) via webinar technology, with the study coordinator on-site to facilitate and distribute materials. A Geriatric Nursing Consultant leads the third session (approximately 30 min) via webinar. The key components of the multifaceted intervention (Table 1) are:

1. Expert opinion leader: Utilizing the framework by Locock *et al.*[55], we define an expert opinion leader as ‘a credible authority (often an academic or consultant) able to explain the evidence and respond convincingly to challenges and debate.’ Such a person is distinct from a peer opinion leader who may be more influential as a role model in daily practice. The expert opinion leader may be particularly valuable in the initial stages of implementing change by ‘translating it into a form which is acceptable to practitioners and takes account of their local experience’ [55]. In our study, the expert opinion leaders are physicians specializing in osteoporosis and/or geriatrics who are active in national/international research and guidelines development.

2. Learning modules: At each session, a learning module is presented and there is opportunity for discussion and active participation. In brief, the first module introduces the study and materials, reviews best practices for OP management and fracture prevention, and provides an orientation to action planning for quality improvement. The second module emphasizes integration of osteoporosis and fracture prevention into care processes, reviews barriers and facilitators, shares strategies from other intervention homes, and provides a case-study exercise. The third module reviews accomplishments and action plan progress, provides information on hip protectors, identifies internal champions, and discusses post-study sustainability including an orientation to resources on our website (http://www.osteoporosislongtermcare.ca[54]).

3. DVD: At the first interactive educational session, the 10-minute ‘Meeting the Challenge of Osteoporosis and Fracture Prevention’ DVD is viewed, and a copy is left at the LTC home so that other staff and residents/families can be educated (available at http://www.osteoporosislongtermcare.ca[54]).

4. Performance reports (audit and feedback): Aggregate/facility-level data for vitamin D, calcium, and osteoporosis medication prescribing are presented in a graphical format at each interactive educational session. Reports are based on the previous month’s prescribing and are benchmarked against other ViD*OS* intervention homes. Confidential, individual performance reports are also provided to each physician.

5. Reminders/Point-of-care tools are distributed and discussed in the educational sessions. These tools include the process indicator checklist, treatment alert, and x-ray requisition stamp (summarized in Table 1). Tools were developed in consultation with the pharmacy provider, the *Ontario Osteoporosis Strategy in LTC*[54] steering group, and our pilot LTC home.

6. Action planning: This quality improvement component is built around the plan-do-study-act (PDSA) cycle [56,57]. In brief, the PDSA process engages teams in planning and managing change by breaking goals into manageable chunks, testing ideas and assessing the results in order to better monitor the impact of changes. Some LTC homes may be familiar with the PDSA process from the ‘Long-Term Care Best Practices Initiative’ [12]. After the learning module is presented, PAC teams discuss and complete an action plan work-sheet at sessions one and two to address barriers and identify organizational strategies, process changes, and specific action items for team members. Teams work on implementing action plans and progress is reviewed at the next session. Strategies generated from sessions are shared with other LTC homes.

7. Internal Champions: The concept of an internal champion is introduced at the first session. By the end of the study it is anticipated that each home will have an internally nominated champion, such as the consultant pharmacist and/or Director of Care. Champions will be linked by a forum on our website (http://www.osteoporosislongtermcare.ca[54]) to facilitate the ongoing sharing of experiences in implementing evidence-based changes in processes of care after the study is completed. Previous studies have found that the sharing of practical tips among LTC homes is useful in the implementation of protocols to improve processes of care [58].

**Table 1:** **Key Components of the Multifaceted ViD *OS *intervention**

Interactive Educational Sessions	Presentation of three learning modules that include a summary of best practices, special considerations for assessing/treating the elderly, key messages, integration of OP/fracture prevention into care processes, and a case study. Opportunity for discussion and active participation via problem-based learning and completion of action plans.
Expert Opinion Leader/ Study Coordinator	Expert opinion leader leads first and second interactive educational sessions via webinar (with study coordinator facilitating on-site). A Geriatric Nursing Consultant leads the third interactive educational session via webinar.
DVD	A 10-min DVD, ‘Meeting the Challenge of Osteoporosis and Fracture Prevention,’ is viewed at the first interactive educational session (and a copy left for the team to educate other/new staff members, residents, families). This DVD was created by the * Osteoporosis Strategy for LTC* (http://www.osteoporosislongtermcare.ca[54]) and is specific to the LTC context.
Performance Reports (Audit and Feedback)	Performance reports for vitamin D, calcium, and osteoporosis medications prescribing (aggregated for all residents in a home) and benchmarked against other ViD*OS* intervention homes, are presented at each interactive educational session. Confidential, individual performance reports are also provided to each physician.
Reminders/Point of Care Tools	Treatment Alert: A tool used by consultant pharmacists to alert physicians and nurse practitioners to assess and consider osteoporosis treatment for residents at increased risk for fracture (based on the 2010 Osteoporosis Canada Clinical Practice Guidelines [21].
	X-ray Requisition Stamp: A stamp labeled ‘please rule out vertebral fractures’ to add to chest X-ray requisitions.
	Process Indicator Checklist: This tool assists teams with creating internal processes and policies that support and sustain appropriate prescribing and other osteoporosis and fractures best practices (*e.g.*, admission/quarterly assessment, diagnoses documentation, ongoing staff education and training).
Action Planning for Quality Improvement	Discussion/completion of a work-sheet to set specific action items for team members, address barriers and facilitators, and outline best practices strategies. Homes work on action plans between study sessions.
Internal Champion	A PAC team member, such as consultant pharmacist and/or Director of Care, who will network with champions at other LTC homes (via online forum http://www.osteoporosislongtermcare.ca[54]) and continue to promote best practices after the research team has left.
Tool-kit*	The tool-kit includes: the 10-min DVD (‘Meeting the Challenge of Osteoporosis and Fracture Prevention’), informational pocket cards, case studies, and posters. Distributed to all LTC homes as part of the *Ontario Osteoporosis Strategy for LTC*.
Osteoporosis Long-Term Care Website*	The website (http://www.osteoporosislongtermcare.ca[54]) with an interactive forum for Internal Champions, is promoted to all LTC homes via the *Ontario Osteoporosis Strategy for LTC.*

#### Control homes

Control homes will receive no intervention. After the intervention homes have completed the study, the control group will have the option of attending a group webinar that presents a summary of key messages from educational modules. They will also be provided with an opportunity to appoint an internal champion who will receive post-study resources and updates. All LTC homes (control and intervention) will also have the opportunity to access continuing education through professional meetings (*e.g.*, the Ontario Long-Term Care Association and the Ontario Long Term Care Physicians).

#### LTC osteoporosis toolkit and website

Both control and intervention homes received the LTC Osteoporosis Toolkit in 2009/2010, which includes a 10-minute DVD, ‘Meeting the Challenge of Osteoporosis and Fracture Prevention,’ pocket cards, case studies, and posters. This toolkit was distributed to all Ontario LTC homes as part of the provincial government-funded *Ontario Osteoporosis Strategy*[59]. The toolkit was developed to increase awareness of best practices for osteoporosis and fracture prevention. An introductory group webinar was available to all LTC homes in Ontario to introduce the toolkit components. In late 2011, the *Ontario Osteoporosis Strategy for LTC* website (http://www.osteoporosislongtermcare.ca[54]) was launched and was promoted in all LTC homes across Ontario.

#### Post-study sustainability

The final phase of the knowledge to action (KTA) cycle [60] includes building sustainability mechanisms into the intervention. It is anticipated that implementation of osteoporosis best practices will continue through the work of each LTC home's internal champion, who will be provided with updated resources and support by a forum on our website (http://www.osteoporosislongtermcare.ca[54]). This will enable the internal champions to interact with each other and share successes and challenges. We will work with our partner pharmacy provider to distribute prescribing reports to LTC homes one year after completing the study. This feedback is important given the high turnover of residents in LTC resulting in a different cohort of residents and staff since the study began.

### Data collection

#### Facility-level

Feasibility data are collected by the study coordinator. Other facility-level data being collected include the number of resident beds, location/type of population centre (*i.e.*, small, medium, or large population centres as defined by Statistics Canada) [61], profit status (profit, non-profit), chain affiliation, and number of treating physicians at baseline.

#### Patient-level

Figure 2 provides an overview of the data collection time-line. De-identified clinical data including demographic, prescribing, and co-morbidities (from the Medication Administration Record) are collected from the pharmacy database by the Director of Systems Services at Medical Pharmacies (JBS).

Falls and fractures data for every resident are collected by a LTC staff member at each home for three-month periods at three times during the study (coinciding with prescribing data pulls, Figure 2). The information source used to populate the data collection spreadsheets may vary by LTC home and sources are documented by the study coordinator. With the recent wide-scale implementation of the RAI-2.0 assessments across Ontario LTC homes [34,35], future studies collecting information on falls and fracture data will likely use this as a data source. The RAI-2.0 is a standard assessment using common methodology and measures and is completed by trained assessors (typically within 14 days of admission and then on a quarterly basis).

#### Qualitative data

After intervention homes have completed the study, individual interviews will be conducted with selected participants to better understand their experience with the intervention. A research assistant (not affiliated with the study) will conduct interviews with two PAC team members (physician and the Director of Care or consultant pharmacist) at approximately seven to ten intervention home sites. Organizational changes to policies and processes in intervention homes will be measured by surveying the Directors of Care regarding the number of items on the process indicator checklist (Table 1) they initiated during the study and by examining changes captured in the action plans.

#### Trial management

The coordinating centre for the study is at McMaster University. The study coordinator and research assistants are responsible for submitting research ethics applications, scheduling of homes, travel arrangements, developing presentations, obtaining and storing consent forms, tracking and recording all decisions and transformations of data made throughout the investigation, and budgeting. All databases are password protected and kept on a secure network system.

#### Data monitoring

No formal comparison between control and intervention homes will occur until the end of the study when final analyses are performed. In accordance with Food and Drug Administration recommendations [62], because the elderly are considered a ‘potentially fragile population,’ a Data and Safety Monitoring Board (DSMB) with expertise in geriatric medicine and clinical trials research will meet to monitor ongoing trial processes. There are no formal stopping rules because the intervention is targeted at health professionals (*i.e.*, does not intervene directly with residents) and because the study is primarily designed to assess feasibility.

#### Blinding

Study participants, personnel, expert opinion leaders, and the analyst who provides the audit and feedback reports are not blinded to home allocation status. The outcome assessor (JBS) who downloads the demographic, prescribing, and co-morbidity data and the analyst who performs the final data analysis will be blinded to home allocation status. The staff members within each LTC home who are recording falls and fractures data are not blinded.

### Data analysis

#### Quantitative analysis

Data from the trial will be analyzed and reported in accordance with the CONSORT criteria [41,63,64]. The baseline characteristics will be reported as mean (standard deviation) or median (minimum, maximum) values for continuous variables and as counts (percent) for categorical variables. The primary feasibility outcomes will be analyzed using descriptive statistics expressed as percent and corresponding 95% confidence intervals (CI). Our primary analyses will be performed using the intention-to-treat principle. The generalized estimating equations (GEE) technique, assuming an exchangeable correlation structure [65], will be used to determine differences between groups for the proportion of residents prescribed vitamin D, calcium and other osteoporosis medication, and number of fractures or falls. The GEE method will take into account the clustered nature of the data, given that residents treated within a LTC home are expected to be similar or correlated (clustered variable will be the LTC home). For the model, the unit of analysis will be the resident and the unit of inference will be the home. There is an increased risk of imbalance at the resident-level because of the home-level randomization. Therefore, resident baseline characteristics that will be included in this analysis are: age, gender, co-morbidities, and the number of prescribed medications. Unadjusted and adjusted odds ratios (OR) and corresponding 95% CIs will be reported. All statistical analyses will be performed using the SAS/STAT 9.2 software package (SAS Institute Inc., Cary, NC, USA) with the criterion for statistical significance set at α ≤0.05.

#### Qualitative analysis

All interview data will be audiotaped and then transcribed verbatim by a professional transcriber. Data analysis will take place concurrently with data collection so that any emerging themes can be incorporated into the interview guide and the codebook. To ensure that we reach informational saturation (all emerging themes are well understood and supported by ample data), any topics that are still unclear after completing the interviews will be revisited in brief telephone conversations with interviewees from LTC homes that provided the least amount of information on those particular topics. A qualitative data management and retrieval software program (QSR-Nvivo) [66] will be used to assist with data organization and retrieval during thematic framework analyses. Both the development of the interview guides and the thematic analyses applied to the transcripts will be guided by two theories that address the change process at the individual and organizational levels: the Theory of Planned Behaviour [67,68], and the Diffusion of Innovations [69-71].

#### Sample size

Our sample size was calculated to detect a difference in the percentage of residents prescribed ≥800 IU/daily vitamin D at follow-up in the intervention versus control groups. We assumed an average of 120 residents per LTC home and that 30% of residents were prescribed ≥800 IU/daily vitamin D at baseline [33]. We postulate a 20% increase in vitamin D prescribing in the intervention group and a 5% increase in the control group (to account for the potential impact of other province-wide initiatives such as the *Ontario Osteoporosis Strategy for LTC*). Based on these assumptions, to detect a 15% difference in prescribing between the groups with an intracluster correlation of 0.10 (two-sided test with significance = 0.05), a sample size of 2,160 residents from 18 LTC homes in each of the intervention and control groups is required to achieve 82% power. Factoring in a potential 10% dropout rate, the recruitment target was 40 LTC homes (20 = intervention, 20 = control).

#### Ethical considerations

The study was approved by the Hamilton Health Sciences/McMaster University Faculty of Health Sciences Research Ethics Board. A representative from the home provided initial consent prior to randomization, and each PAC team participant provides written informed consent at the first educational session.

## Discussion

KT research in LTC is still in its early stages [4-6]. Our project is one of the first RCT studies to examine the effectiveness and feasibility of a multifaceted, interdisciplinary KT intervention in LTC. Given that this is a pilot RCT, feasibility measures such as recruitment and retention, attendance at educational sessions by PAC members, and use of study materials are important outcomes for planning future interventions. Furthermore, results from the upcoming qualitative phase of the pilot study will provide valuable information about the KT-related needs of health professionals working in LTC. It will also assist us in gaining some preliminary data on what may be the most active ingredients of the complex intervention. A better understanding of these factors will enable future researchers and care providers to select and tailor KT strategies that can maximize the uptake and utilization of evidence in LTC.

Increasing the utilization of available evidence by clinicians in daily practice is difficult in any setting, and the LTC environment presents some additional unique challenges. Prescribing for the frail elderly is especially challenging due to the presence of co-morbid illness, frequently large number of medications, functional impairment, cognitive deficits, and age-associated decline in renal function [72-74]. The majority of practice guidelines do not adequately address the challenges of applying recommendations to elderly patients, particularly those with co-morbidities [75]. In addition, the composition and skills of the nursing staff in LTC are different than in other sectors because nurse’s aides provide most of the direct care and their care rituals are often focused on task completion [19].

Implementing evidence-based practices in LTC is made more complex by the fact that the physicians are not typically located at the LTC home. Instead they rely on the on-site healthcare team to inform and update them about their patients' conditions. Clinical leaders are often registered nurses (as opposed to physicians in acute or primary care settings) [76], and they assume greater responsibility for the coordination, decision-making, and administration of drug-related interventions [77].

Heavy paperwork and institutional requirements, limited resource and staffing levels, limited time to implement protocols, the absence of a learning culture, entrenched ways of learning and communicating, ‘change fatigue,’ and high staff turnover are other postulated factors that inhibit the uptake of evidence-based practices [6,20,78,79]. In a study of registered nurses working with the elderly [80], the most commonly noted barriers to research use were the lack of a cadre of knowledgeable colleagues with whom they could discuss research issues, facility-level barriers, the lack of time to read research studies and the fact that research findings are not readily available in a single location. These nurses believed that establishing networks among colleagues, staff, researchers, and physicians would enhance the uptake of research evidence.

If the above challenges can be addressed, there are elements in the LTC environment that also make it conducive to implementing best practices. For example, as noted by Berta *et al.*[6] LTC homes are ‘small, structurally flat, and highly reliant on collaborative decision making’; thus decision makers and staff in LTC may be more amenable to implementing complex innovations than in other practice settings.

The design and implementation of our intervention was founded on the well-known Canadian Institutes of Health Research Knowledge-to-Action cycle [60]. In this paper, we describe how our intervention is adapting knowledge to the local context (LTC homes in Ontario) and continuously assessing barriers and facilitators to knowledge use. Despite the importance of tailoring the intervention based on identified barriers, recent evidence suggests that many studies do not effectively do this [81]. We have tailored the intervention to better meet the needs of LTC care providers by incorporating our educational sessions within regularly scheduled PAC team meetings, developing the reminders and point-of-care tools in partnership with front-line LTC providers, and engaging staff in identifying their own site-specific strategies needed to address barriers (*e.g.*, action planning). We are monitoring knowledge use throughout the study via the performance reports and action plans completed on site and evaluating outcomes using quantitative, qualitative, and process measures. Through ongoing work with internal champions, our partner pharmacy provider, and our website forum (http://www.osteoporosislongtermcare.ca[54]), we have built in sustainability mechanisms. A Swedish study [18] that examined whether nurses who continued continuous quality improvement (CQI) activities over several years emphasized that supportive leadership and access to individuals with research expertise were key factors in sustained evidence-based practice.

Our design elements are also congruent with recommendations from the Nursing Home Quality Initiative launched in 2002 by the Centers for Medicare and Medicaid Services in the United Stated [58]. Lessons learned about implementing CQI in LTC included: forming partnerships with LTC stakeholders, engaging physicians and Medical Directors in the CQI process, teaching CQI principles to all LTC staff, facilitating the exchange of successful strategies and practical tips among LTC homes, and providing one-on-one assistance to LTC staff and administrators. Frequent contact with and involvement of the entire CQI team were identified as essential to overcoming problems stemming from high staff turnover and heavy workload demands on Administrators and Directors of Nursing [58]. ViD*OS* has taken this advice and worked closely with the entire PAC team, including Medical Directors, to develop and update action plans to improve bone health of LTC residents.

Certainly one of the frequently asked questions regarding multifaceted interventions is: which components of the ‘black-box’ are most effective? Although we cannot answer this question quantitatively, we will attempt to address this issue in the qualitative phase of the study by asking participants how effective they perceived the various elements of the ViD*OS* intervention to be, and what individual and organizational factors they believe facilitated or inhibited the change process.

The lessons learned from this pilot RCT will be helpful when planning future KT research on other health issues in LTC settings. This includes insights on the KT process (*e.g.*, recruitment and retention of leaders in innovation), resources (time and budget issues), management (personnel and data management issues), and scientific evidence (effect sizes, intracluster correlation) [42]. It is anticipated the final results of this study will be presented in 2013.

## Abbreviations

(ViD*OS*): Vitamin D and Osteoporosis Study; (LTC): Long-term care; (KT): Knowledge translation; (PAC): Professional advisory committee; (CIHR): Canadian Institutes for Health Research; (CQI): Continuous Quality Improvement; (KTA): Knowledge to action; (PDSA): Plan Study Do Act; (RCT): Randomized Controlled Trial.

## Competing interests

Alexandra Papaioannou is or has been a consultant, or on a speaker’s bureau, or received unrestricted grants for Amgen, Eli Lilly, Merck Frosst Canada, Novartis, Warner Chilcott; she also has conducted clinical trials for Eli Lilly, Merck Frosst, Novartis and Pfizer.

Jonathan D. Adachi is or has been a consultant, or on a speaker’s bureau for Amgen, Eli Lilly, GSK, Merck, Novartis, Pfizer, Procter & Gamble, Roche, Sanofi Aventis and Warner Chilcott; he has also conducted clinical trials for Amgen, Bristol-Myers Squibb, Eli Lilly, Merck, Novartis, Pfizer, Procter & Gamble, Sanofi Aventis, Roche and Warner Chilcott.

Suzanne Morin has received an unrestricted research grant from Amgen, as well as honoraria for consultation work and presentation from Merck, Eli Lilly, Amgen, Novartis and Warner Chilcott; she is also a chercheur-clinicen boursier des Fonds de Recherche en Sante du Québec.

Sharon Marr has received unrestricted educational funds from Merck Canada Inc., Amgen, Lilly, Lundbeck, Medical Pharmacies Group Ltd., Novartis.

Robert Josse is on the advisory board or have received research grants or speaker honoraria from Merck, Lilly, GSK, Warner Chilcott, Amgen, Novartis.

Courtney C. Kennedy, George Ioannidis, Lora M. Giangregorio, Lehana Thabane, Richard G. Crilly, Lynne Lohfeld, Laura E. Pickard, Susanne King, Mary-Lou van der Horst, Glenda Campbell, Jackie Stroud, Lisa Dolovich, Anna M. Sawka, Ravi Jain, Lynn Nash declare that they have no competing interests.

## Authors’ contributions

AP conceived of the study and contributed to the design and implementation, oversaw its coordination, participated in the delivery of the intervention sessions, and helped to draft the manuscript. CCK contributed to the conception, design and implementation of the study, participated in its coordination, performed data analysis and interpretation, and drafted the manuscript. GI contributed to the conception, design, and implementation of the study, performed data analysis and interpretation, edited the manuscript for important intellectual content, and gave final approval of the version to be published. LMG contributed to the conception, design, and implementation of the study, edited the manuscript for important intellectual content, and gave final approval of the version to be published. JDA, SNM, RGC, SM, RGJ, and MLV contributed to the conception and design of the study, participated in the delivery of the intervention sessions, edited the manuscript for important intellectual content, and gave final approval of the version to be published. LT contributed to the conception and design of the study, provided consultation regarding RCT methodology, edited the manuscript for important intellectual content, and gave final approval of the version to be published. LL contributed to the conception and design of the study, provided consultation regarding qualitative methodology, edited the manuscript for important intellectual content, and gave final approval of the version to be published. SK, LEP, contributed to the conception, design, and implementation of the study, coordinated the study, edited the manuscript for important intellectual content, and gave final approval of the version to be published. GC contributed to the conception, design, and implementation of the study, participated in data acquisition, edited the manuscript for important intellectual content, and gave final approval of the version to be published. JS contributed to the conception and design of the study, coordinated data acquisition, edited the manuscript for important intellectual content, and gave final approval of the version to be published. LD, AMS, RJ, and LN contributed to the conception and design of the study, edited the manuscript for important intellectual content, and gave final approval of the version to be published. All authors read and approved the final manuscript.
